# Supporting Transgender Inclusion and Gender Diversity in Schools: A Critical Policy Analysis

**DOI:** 10.3389/fsoc.2020.00027

**Published:** 2020-04-23

**Authors:** Kenan Omercajic, Wayne Martino

**Affiliations:** ^1^Faculty of Education, University of Western Ontario, London, ON, Canada; ^2^Faculty of Education, University of Western Ontario, London, ON, Canada

**Keywords:** gender justice, gender democratization, trans-affirmative policy, transgender, trans inclusion, gender diversity, transgender students, non-binary

## Abstract

In this article, we conduct a policy analysis of transgender affirmative policies in Ontario and examine their implications for addressing gender justice and gender democratization in the school system. By adopting a case study approach, we provide a critical analysis of these policies and of how stakeholders with familiarity and knowledge of trans-affirmative policies from two school boards in Ontario are making sense of their impact with respect to addressing trans inclusion in schools. As such, our study offers insight into two trans-affirmative policies and their implications for both supporting transgender, gender non-conforming and non-binary students and envisioning gender-expansive education in the school system. We draw on interviews with key informants—two teachers and a school board official—as a basis for reflecting on the need to move beyond a discourse of accommodation in trans inclusive policies to one that explicitly articulates a pedagogical commitment to gender justice and gender democratization in schools.

## Introduction

In this article we provide a critical analysis of trans-affirmative policies from two school boards in Ontario and examine their implications with respect to supporting transgender and gender diverse students in the education system. This focus is important in light of the high rates of harassment, victimization, absenteeism, and suicide among transgender and gender diverse youth in schools that are documented in the existing literature (Wyss, 2004; Greytak et al., 2009; Taylor et al., 2011; Egale, 2012; Human Rights Campaign Gender Spectrum, 2018). Our purpose is to generate knowledge and understanding about how to best support trans and non-binary youth in schools by undertaking a critical policy analysis that addresses the limits of accommodation and the necessity of embracing gender democratization through pedagogical and curricular intervention (Youth Gender Action Project, 2009; Martino and Cumming-Potvin, 2016, 2019; Smith and Payne, 2016; Frohard-Dourlent, 2018; Luecke, 2018). Hence, our study contributes to an emerging body of trans-focused scholarship that is concerned to address gender diversity and transgender inclusion in the education system (Greytak et al., 2009; Ryan et al., 2013; Millei and Cliff, 2014; Payne and Smith, 2014; Frohard-Dourlent, 2016; Bartholomaeus and Riggs, 2017; Ullman, 2017; Goodrich and Barnard, 2018; Leonardi and Staley, 2018; Sinclair and Gilbert, 2018; Slater et al., 2018; Carlile, 2019; Kjaran, 2019; Martino and Cumming-Potvin, 2019). Firstly, we outline our approach to critical policy analysis and explicate a trans-informed framework for understanding our approach to addressing gender diversity in the education system more broadly before examining the particular school board policies in question. We then go on to present the viewpoints of three key informants who provide further insight into these policies and trans inclusion in schools.

## Framing Approach to Policy Analysis

We initially consider these policies through Bacchi's lens of problematization and “the way in which the ‘problem' is represented [which ultimately] carries all sorts of implications for how the issue is thought about and for how the people involved are treated” (Bacchi, 2009, p. 1). Bacchi's (2009) approach builds upon Foucauldian principles of subjectification and inquires how the subject comes to be constructed and constituted through policy (p. 16). She argues that in conceiving of policy as discourse the “emphasis … is upon the ways in which language, and more broadly, discourse sets limits upon what can be said” (Bacchi, 2000, p. 48). Bacchi further elaborates that this approach to critical policy analysis is about “recogniz[ing] the non-innocence of how ‘problems' get framed within proposals, how the frames will affect what can be thought about and how this affects possibilities for action” (p. 50). Hence, we are concerned to draw attention to the limits of how specific school board policies *construct* the problem of the trans subject as an object of intervention with respect to articulating specifically the conditions necessary for supporting trans youth, and more broadly, gender diversity in the education system. It is the *policy frames* informing the production of these texts, which rely on a fundamental logics of accommodation as a basis for addressing the problem of the need for trans inclusion, that is a critical focus in our analysis of these texts.

Relatedly, we also draw on Ball's (1993) framing of “policy as text” with its interpretive repertoires that are products of multiple agendas and compromises which are enmeshed in networks of governance with their specific contingencies and shifting conditions of emergence. For example, given that policies are (multi)authored and read and enacted in a variety of settings, it is important to understand that: “Few policies arrive fully formed and the process of policy enactment also involve ad-hockery, borrowing, re-ordering, displacing, making do and re-invention […] The onus is on schools to ‘make' sense of policy where (sometimes) none is self-evident” (Ball, 1993, p. 8). Moreover, each stakeholder may interpret a policy differently, and so the written text does not necessarily result in the same actions being undertaken by each school. Important questions related to how policies are read and interpreted, their priority, the environment they enter, and the motivation of stakeholders to enact them need to be considered. Hence, in this article we investigate how several stakeholders with familiarity and knowledge of trans-affirmative policies within the context of their respective school board/school are making sense of these policies, and how their insights might be utilized to further inform possibilities for addressing trans inclusion and gender diversity in the education system.

Overall, we underscore the importance of *policy as discourse*, which considers not only what policymakers choose to incorporate in policy, but also that which they do not think about or *deliberately* choose to exclude, underscoring that policy is not simply just text, but embedded in the exercise of power through “a *production* of truth and knowledge, as discourses' (Ball, 1994, p. 21). As such we draw attention to the ways in which policy texts constitute the terms of trans inclusion and support for transgender youth in schools and how such texts are interpreted by key stakeholders such as educators in schools, who are the targeted recipients of these policies. In this regard, we investigate the extent to which transgender inclusion and support for trans youth are understood to be “spoken by policies” (Ball, 1994, p. 22).

## Trans-Informed Theoretical Frameworks

We draw specifically on trans-informed theoretical frameworks which inform both our understanding of trans inclusion and policy governance. For example, Spade (2015), trans law scholar and activist, argues that attention needs to be directed to the administration of trans polices rather than focusing just on the “law” or policy itself as a basis for investigating their impact with respect to addressing trans inclusion and gender diversity. This focus on the administrative aspects of governance does not deny the need for human rights legislation and policy development, but rather, directs attention to learning more about how such policy frames relate or rather translate into enhancing “trans well-being” and gender diversity in schools (Ashley, 2018, p. 1). Indeed, Spade advocates for a shift in focus from an individual human rights framing of discrimination to one that addresses more broadly regimes of gender classification and categorization: “Such a shift requires us to examine how administrative norms or regularities create structured insecurity and (mal)distribute life chances across populations” (p. 9). In this respect, as part of our case study we provide a snapshot into how three key informants are making sense of the policy and what the implications are for creating spaces in schools for addressing trans marginalization and gender expansive education (Ullman, 2017; Cumming-Potvin and Martino, 2018).

Such a trans-informed analytic perspective is important as it has the capacity to inform our understanding of how transgender inclusivity and gender diversity are being considered in education policy contexts, with implications for addressing the erasure and invisibility of trans lives. This focus is necessary given Namaste's (2000) explanation that erasure is “a defining condition” of trans people's lives (p. 4). In fact, Stryker (2006), argues for a Transgender Studies focus that addresses

anything that disrupts, denaturalizes, rearticulates, and makes visible the normative linkages we generally assume to exist between the biological specificity of the sexually differentiated human body, the social roles, and statuses that a particular form of body is expected to occupy, the subjectively experienced relationship between the gendered sense of self and social expectations of gender-role performance, and the cultural mechanisms that work to sustain or thwart specific configurations of gendered personhood (p. 3).

Hence, we are interested in understanding how a trans-informed critical analysis might be interwoven in trans-affirmative policies, along with a commitment to addressing the impact and effects of “assumptions regarding sex and gender, biology and culture…” (Stryker and Aren, 2013, p. 3).

This commitment entails unpacking the ways in which gender and non-binary classifications are administratively addressed in these policies to better understand how they perpetuate or minimize the “vector of violence and diminished life changes” for transgender and gender diverse youth in schools (Spade, 2015, p. 142). As such, we examine how the recognition of transgender personhood and its livability are understood within the limits and possibilities that are circumscribed by trans-informed policies in the school system that rely on a fundamental logics of accommodation. What knowledge about transgender phenomena and gender diversity are articulated through such trans-inclusive policies and what are their implications for ensuring gender justice and democratization in the education system (Connell, 2009; Martino and Cumming-Potvin, 2018)?

Such trans-informed frameworks on gender democratization require a critical focus on the impact of *cisgenderism* and cisnormativity in schools which “refers to the cultural and systemic ideology that denies, denigrates, or pathologizes self-identified gender identities that do not align with assigned gender at birth” (Lennon and Mistler, 2014, p. 63). These cisnormative regimes of practice also reinforce what Rands (2009) refers to as the *gender oppression matrix*, which involves privileging individuals who conform to gender norms while punishing those who transgress them. As such, Rands advocates for the need to embrace gender complex frameworks in ways that complement the trans epistemological underpinnings of this study (Stryker, 2006; Martino and Cumming-Potvin, 2018). In light of this framing, we acknowledge that institutionalization of cisgendersim in schools contributes to a cultural hegemony which privileges certain gender identities and forms of embodiment over others (Spade, 2015; Nicolazzo, 2017c). As Connell (2009) argues, there is a need to confront gender hierarchies and their effects which she envisions as a commitment to *gender democratization* (p. 146). In this respect, gender democratization moves beyond the discourse of trans inclusivity that relies solely on a fundamental logics of accommodation and liberal notions of human rights to address curricular and pedagogical reform that accounts for more expansive and equitable understandings of gender (Courvant, 2011; Martino and Cumming-Potvin, 2015, 2018; Keenan, 2017).

## About the Study

We chose to focus on two school boards which were the first to develop trans-affirmative policies in Ontario and conducted semi-structured interviews with one policymaker and two educators familiar with such policies. These school boards fall under provincial jurisdiction whereby each province in Canada is responsible for creating its own educational structures^1^. Canada's constitution, known as *The Constitution Act of 1867*, stipulates that “[I]n and for each Province the Legislature may exclusively make Laws in relation to Education…” (s. 93) ^2^. Legislation consists of provincial statutes, along with bylaws and regulations of local school boards or commissions that set out the division of responsibilities in the area of public instruction. School Board A is one of the largest and most diverse in Canada, covering a large urban center and serving a school population of over 200,000 students. It was the first school board in the country to develop a trans-affirmative policy in 2011, prior to the Ontario Human Rights Commission authorizing the inclusion of gender identity and gender expression as legislative grounds for discrimination in 2012, underscoring a commitment to addressing trans inclusivity and gender diversity in their schools. School Board B is a smaller board serving urban, suburban and rural communities in Ontario with a population of over 74,000 students, which introduced a trans-affirmative policy in 2012. Firstly, we provide an overview of these policies utilizing a trans-informed lens in analyzing their specific discursive articulations of trans-inclusivity. We reflect on the insights regarding the limits of accommodation in trans-affirmative policy gleaned from the interviews with key informants from each of these boards. In this sense, our approach to analyzing these policies was informed by both Bacchi's WPR ('What's the Problem Represented to Be?') approach, and Stephen J. Ball's interrogation of policy as *text* and policy which draw attention to the interpretive aspects of highlighting the discursive frames of accommodation that come to define the limits of how trans-informed understandings are articulated for schools. As such our overall critical analysis is specifically informed by our engagement with trans informed theoretical frameworks that draw attention to the need to address more systemic matters related to gender justice involving the institutionalization of cisnormativity.

We employed a case study design with the specific aim of “gather[ing] comprehensive, systematic, and in-depth information” (Patton, 2015, p. 536) about trans-affirmative policies. It was the attention directed not only to the examination of the key roles by policymakers and stakeholders in the creation of these policies, but also the knowledge and perspectives of educators whose understandings of trans inclusivity were guided by their knowledge of these policies that we were concerned to investigate. Inquiring about their interpretive understandings of the policies, therefore, lent itself to embracing a qualitative case study research design (Patton, 2015). In this respect, the study was not conducted with the aim of generalizing about the impact of such policies, but rather to generate knowledge about how such policies articulate understandings about trans inclusions and with what political effects. The focus on key informants through purposive and snowball sampling (Patton, 2015) allowed us to draw on the insights and experiences of the following three participants who had either a hand in creating these texts, or first-hand experiences of their administration: *Grace*, a non-binary individual, had been a high school teacher of visual arts, French and special education with School Board B for the past 5 years; *Dean*, a transgender man, who has been an elementary school teacher in School Board B for 29 years and *Michael*, a cisgender male, with 17 years of administrative experience as an equity officer, who contributed to the development of School Board Policy A. These participants were specifically selected due to their knowledge and experience(s) with the policies we analyzed. They were selected through purposive and snowball sampling measures among the limited pool of administrators/educators who had experience or critical feedback specific to the policies. In this regard, they provided “in-depth knowledge about particular issues” (Patton, 2015, p. 219) and they were selected due to their expertise regarding trans-affirmative policies in their respective school boards. Given the specific nature of case study research, we were not so much concerned to generalize across a population of educators, but to provide an analytic focus on the particularity of the policies in question in light of the existing literature in the field about the barriers to supporting transgender inclusion and gender diversity in schools (Payne and Smith, 2014; Frohard-Dourlent, 2016; Meyer et al., 2016; Morgan and Taylor, 2018). All participants signed a consent form agreeing to both audio recording of the interviews and the non-identifying data being used in research publications. Teacher participants were asked to share their knowledge with respect to the policy in their school board. They were prompted to provide their overall assessment and impressions of the trans-affirmative policy and what impacts—if any—they may have seen in their schools as a result of the policy. Policy creators were asked how the policy came about, why they felt it was necessary, and how effective they believe the policy has been in achieving its purpose.

A thematic analysis of the interview data was undertaken by means of “identifying, analyzing, and interpreting patterns of meaning” as a result of a constant reading and re-reading of the interview transcripts (Clarke and Braun, 2017, p. 297). The significance of policy and curriculum as sites of intervention and the limits of relying on a discourse of accommodation emerged as key themes that further enhanced our own interpretive and critical examination of the trans inclusive policies that are the subject of this article.

## Trans-Inclusive School Board Policies Within the Ontario Legislative Context

In this section, we focus our attention on two specific school board policy texts in question. Trans-informed policy analysis at the local level of school boards in Ontario needs to be understood as a response to broader legislative frameworks at the provincial level. For instance, gender identity and gender expression were included in 2012 as part of the Ontario Human Rights Code (OHRC) (Ontario Human Rights Commission, 2012a). As a result, Ontario became the first province in Canada to legally recognize the term “gender expression” (Kirkup, 2018, p. 109). Conversely, at the federal level there has been a struggle to introduce similar grounds for discrimination. Bill C-16 (2016)—a federal government sponsored bill that prohibits discrimination on the basis of gender identity and gender expression—was introduced into the House of Commons in May 2016. In this sense, it is significant to understand that Ontario, as a province, has been far more progressive in its consideration of trans and gender diversity with respect to law and policy, and as such, so have the provincially governed secular school boards (Martino et al., 2019).

**School Board Policy A** was released in 2012, the first trans specific school board policy of its kind in Canada (see Shanks and Lester, 2019), and offers valuable considerations, ranging from pronoun usage, privacy, and structural accommodation(s) (i.e., all-gender bathrooms and change rooms), with an emphasis on safety and protecting the human rights of trans students. Bacchi (2009) encourages the start of any policy analysis to lead with the question of what the problem is represented to be. In this case, the policy was created “to raise awareness and help protect against discrimination and harassment [and] fulfill a shared obligation to promote the dignity and equality of those whose gender identity and or gender expression does not conform to traditional societal norms.” In order to address this problem, the policy emphasizes the need for accommodation, insisting that its *goal* is to set out “best practices related to accommodation based on gender identity and gender expression.”. The policy relies on a fundamental discourse of accommodation as a basis for both raising awareness about and addressing harassment and discrimination of trans people in the education system. It indicates that schools must address “each student's needs and concerns separately” and states that staff “should not disclose a student's transgender/gender non-conforming status to others” or to “the student's parent(s)/guardian(s)/caregiver(s) without the student's explicit prior consent” unless necessary. This stipulation reflects a legal requirement as set out in the Ontario Human Rights Code with regards to protecting and respecting confidentiality as it pertains to disclosure of one's transgender status (Ontario Human Rights Commission, 2012b). The policy also emphasizes the student's “right to be addressed by a preferred name and pronouns corresponding to their gender identity.” In this capacity, the policy places the “student in charge” in an effort to demonstrate that they are the “driver” of their own narrative (Frohard-Dourlent, 2018, p. 332). However, while the policy endorses agency with respect to pronoun usage, it does not explicitly address how to sustain these reiterative vocalizations of trans and non-binary identification and embodiment which are presented in terms of the individual right of the student to request such forms of address. In this respect, the policy omits the importance of a continued commitment to these reiterative vocalizations and maintains “the power relations that a discourse constructs and allows,” rendering the cisnormative system primarily unchallenged (Ball, 1994, p. 22).

In fact, School Board Policy A seems to envision gender inclusivity to be fundamentally bound primarily to physical accommodation. The policy text sets its focus on the potential for the existence and inclusion of trans and gender diverse bodies in physical spaces, specifically outlining individual procedures to be taken into account with respect to student and staff requests for accommodation. In fact, physical accommodation is foregrounded in the body of the document with its emphasis on students having the right to “safe restroom facilities and the right to use a washroom that best corresponds to the student's gender identity, regardless of the student's sex assigned at birth.” The policy explicitly addresses accommodation in the space of physical education, which is typically gender segregated. Specifically, it insists that staff must ensure that “students can exercise their right to participate in gender-segregated […] class activities in accordance with each student's gender identity.” Accommodation in this area also emphasizes the right of students “to a safe change-room that corresponds to their gender identity.” However, the onus for such accommodation rests with the individual student requesting such a space. Thus, the fundamental discourse of accommodation governing the terms of trans inclusion in this policy is one which constitutes the *individual* trans student as responsible for ensuring their own safety and well-being and requires them to basically be *out* in order to do so. Such a policy stipulation actually flies in the face of research in Canada and elsewhere that shows that trans students are particularly vulnerable to both verbal and physical and harassment. Key findings from a national school climate survey in Canada found that school climate was far more hostile and unwelcoming to transgender students: “[a]lmost three-quarters (74%) of trans students reported being verbally harassed about their gender expression” (Taylor et al., 2011, 23). In fact, this study found that “trans students were more likely to report hearing negative gender-related or transphobic comments daily or weekly from other students (89.8% of trans youth)” (p. 52) (see also Greytak et al., 2009). Concerning physical harassment, Taylor et al. (2011) found that “trans students were much more likely than sexual minority or non-LGBTQ students to have been physically harassed or assaulted because of their gender expression (37.1%)” (p. 64). They also investigated the extent to which schools responded and intervened to instances of transphobia and that school-based policies paid “insufficient attention to the damaging effects of negative gender-related comments on students, especially trans youth, who are most often the target of these remarks” (p. 117). In addition, the study also found that “nearly half (43.0%) of trans students reported that school staff members never intervened when homophobic comments were being made” (p. 110).

School Board Policy A does offer an acknowledgment of the importance of trans-inclusive content in teaching and in all subject areas, including a separate section that addresses “curriculum integration.” It calls for the need to address the erasure of transgender and gender non-conforming individuals from the curriculum which “creates a misconception among many students that transgender people do not exist and are an object of scorn.” The policy also advocates for school board and curriculum-based leaders to “integrate trans awareness and trans positive advocacy training into staff professional development curricula.” Such a consideration is important and underscores Rands' (2009) point that educators must be prepared adequately “to teach gender in more complex ways that take into consideration the existence and needs of transgender people” (p. 419). However, no accountability measures or allocation of resources are actually stipulated to ensure such professional and curricular development. In fact, in order to ensure that educators are equipped to do this, School Board Policy A places responsibility on librarians in schools to “acquire trans-positive fiction and non-fiction books for school libraries and encourage the circulation of books that teach about gender non-conforming people.” If policy as text reflects policy as a product of compromises between different agendas and interests, then this stipulation reads far more as a non-committal compromise or formality rather than a devotion to follow through on pedagogical and curricular development (Ball, 1994, 17). “Different interpretations” (Ball, 1994, 17) of “trans-positive” books and what it means to “encourage the circulation of books” leaves this commitment relatively ambiguous.

In conjunction with this stipulation, the policy text provides an appendix of resources for students and parents, ranging from reading materials (which include handbooks about parenting transgender and gender diverse children), online resources for trans youth and their families, and also identifies support groups for trans youth. In this sense, the policy text is indirectly informed by research which indicates:

Educators, policymakers, and safe school advocates must continue to seek to understand the specific experiences of transgender students, and implement measures to ensure that schools are safe and inclusive environments for all LGBT youth. Given the potential positive impact of supportive educators, student clubs, curricular resources, and comprehensive anti-harassment policies on the school experiences of LGBT students, it is imperative that schools work to provide these resources to students. Along with providing access to LGBT-related resources, it is important for educators, advocates, and policymakers to recognize how the needs of transgender youth may both be similar to and different from the needs of their non- transgender peers. Schools should explicitly address issues and experiences specific to transgender students (Greytak et al., 2009, p. 54–55).

However, there is no explicit attention to addressing the institutionalization of cisgenderism as part of a boarder commitment to the educative “work that must be done to create classrooms that truly integrate trans lives into current curricula and classrooms” (Courvant, 2011, 26; Malatino, 2015;Keenan, 2017).

Nevertheless, most of these practical examples and resources are reserved for the appendices in the policy text, while more general assertions and assurances about curricular inclusion are reserved for the main text, as outlined above. As a result, there is an absence of any explication of how a trans-inclusive pedagogy and curriculum might be enacted or any specific allocation of resources to achieve such outcomes (Keenan, 2017). This is an important policy consideration for, as Nicolazzo (2017a) expresses, “just as trans^*^ people need physical space to be themselves, we also need epistemological spaces of our own to learn how we come to know ourselves and our worlds through gendered perspectives” (p. 7). In this respect, trans-specific policies need to move beyond a discourse of policies *for* transgender individuals toward policies that engage with them and constructively consider how such integrations can restructure a cisgenderist system in light of the provision of necessary supports and resources for principals and schools to ensure that gender expansive education can be enacted (Mangin, 2018).

**School Board Policy B**, published in 2013, is closely modeled on and was adapted from School Board Policy A. However, instead of taking the opportunity to build on its predecessor, School Board Policy B copies word for word entire sections that are lifted directly from the School Board Policy A with some alterations and omissions. In fact, one of the creators of School Board Policy A was consulted to help draft School Board Policy B, likely due to the fact that the former school board is known for its reputation as a leader in equity and social justice education.

The replication of policy documents from one context to another emphasizes the act of what Phillips and Ochs (2004) refer to as policy borrowing, which is understood as the “conscious adoption in one context of policy observed in another” (p. 774). It also highlights the board's disengagement from the understanding that policy practices are “specific and contextualized” and are “framed by the ethos and history of each school and by the positioning and personalities of the key policy actors involved” (Braun et al., 2010, p. 558). This act of policy borrowing is evident from the introductory page of School Board Policy B, where the policy uses the same excerpt from the Ontario Human Rights Code as School Board Policy A to detail the significance of providing “equal rights and opportunities, and freedom from discrimination.” This insertion highlights the exigency behind the need to respond to provincial legislation for ensuring the rights of gender minorities in public and state funded institutions. In this respect, there is a necessity for policy networks to coincide and value intersecting identities that endure discrimination and not just one group; this reliance on the OHRC as a foundation must go further in underscoring the importance of intersectional identities and how these multiple vectors invite further issues of harassment, discrimination and trans marginalization (Spade, 2015). Moreover, the policy mirrors the representation of the problem as outlined by School Board Policy A, suggesting that it has been “designed to raise awareness and help protect against discrimination and harassment.” While the problem presented (Bacchi, 2009) is understood in terms of ensuring protection from harassment and discrimination, such a commitment is understood as enacting accommodation measures at the request of the actual trans student. Ironically such a policy stipulation puts the student in the *driving seat* for ensuring their own accommodation with no specific accountability being required for the actual system to take responsibility for trans inclusive interventions. Frohard-Dourlent (2018), e.g., argues that trans inclusive policies and practices which rely on student led reform agendas are limited and that what is required is the need for more systemic driven approaches that “do not require the presence of trans bodies and instead offer possibilities for educational spaces in which all students would experience fewer pressures of gender and sexual conformity” (p. 328).

School Board Policy B also borrows the section “accommodation based upon request” directly from School Board Policy A, but with significant omissions, speaking to how policy as discourse emphasizes constraints imposed by discourse through the purposeful omission of select sections of text (Ball, 1994; Bacchi and Eveline, 2010). Specifically, School Board Policy B does *not* highlight that “there is no age limit on making an accommodation request” or the suggestion to put a request “in writing for purposes of clarity” and protection, which begs the question of what the purpose of this intentional omission might entail. However, it *does* offer specificity with respect to contingency when it comes to unresolved requests and outlines how both students and employees can respectively “seek recourse” if they feel that their accommodation needs remain unmet. School Board Policy A does not offer such a potential to appeal accommodation measures. However, much like School Board Policy A, it continues to place the student in charge of their own accommodation(s), assuming “that students have the power and language to assert individual needs and identify solutions to potential conflicts” (Frohard-Dourlent, 2018, p. 338).

It is significant that the section on trans curricular development and integration included in School Board Policy A is *omitted* from the School Board Policy B text. Such an omission reflects an *active* decision not to address, explicitly, the curricula necessity for elaborating the terms of what a “gender-complex education” might entail with its emphasis on ensuring that educators and students are incessantly “aware of the ways in which the gender oppression matrix and heterosexism work in tandem to privilege certain groups of people and oppress others and take action to challenge the gender oppression matrix and heterosexism” (Rands, 2009, p. 426). By actively removing this piece from the policy, the policy itself reaffirms a regime of truth in which support for challenging dominant, cisgender discourses with respect to the provision of gender expansive education is not specifically addressed. This aspect of what Ball (1994) refers to as “the processes of policy influence and text” highlights that “only certain influences and agendas are recognized as legitimate” (p. 17): “Policies do not normally tell you what to do, they create circumstances in which the range of options available in deciding what to do are narrowed or changed, or particular goals or outcomes are set” (p. 19).

In addition, while School Board Policy A encourages school libraries to include books and resources that deal with gender diversity, this detail is *also* removed from and not acknowledged in School Board Policy B. Dean, a trans educator, noted that even when libraries do contain materials discussing gender diversity, they are not always visible nor physically attainable:

I do know that my perfectly well-meaning, sweet, friendly librarian who used to be at my school would hit books that were about “sensitive topics,” like gay things, really high up so that none of the children could get at them. I mean, you now, the younger children. Maybe the [grade] 7's and 8's might be able to reach them, if they looked in that area. It's like we're putting them up without putting them up. We'll put them up and never ever talk about them or encourage anybody to look over there.

Dean's insight demonstrates the lack of consideration—both in policy and practice—of how trans and gender diverse students can exist epistemically and need to see themselves reflected within the education system (Nicolazzo, 2017a; Martino and Cumming-Potvin, 2018), nor does the policy account for statistics that indicate that the inclusion of such resources minimizes rates of victimization (Greytak et al., 2009). Despite removing these curricular considerations from their replicated policy text, School Board Policy B includes the same appendix about making schools safer and more gender affirming places, without acknowledging how a trans-inclusive curriculum can contribute to achieving these goals (Taylor et al., 2011).

Dean accounts for the difficulty of attaining resources that address gender diversity. While Prosser (1998) notes that examining transgender narratives in curriculum will result in introducing a more expansive discussion of gender and gender embodiment, leading to a deeper understanding of the spectrum of identities. Dean, however, noted that lack of access to trans-affirmative materials and resources, despite occasional efforts by the Ministry of Education, serves as a great barrier to properly implementing a trans-inclusive curriculum:

That's always the thing where you say curriculum and materials, what can we come up with? And so, there are novels that we can find. And there's been some great stuff written by ETFO [The Elementary Teacher's Federation of Ontario], you know? […] But what happens is that the books… they have a very short market time… when they're on queer topics. And so, you make this whole lesson plan or whatever it is… Resources based on this book, and then you can't get a hold of the book!

In this respect, while resources may be recommended or listed in policy appendices for teachers to access in order to be inclusive in their pedagogy, acquiring these resources proves particularly difficult in practice. Such a lack of access suggests that although policies are created to be inclusive and to accommodate trans students, and to even “raise awareness,” such efforts fall flat due to a lack of investment in resources and tools for curricular intervention. Despite the fact that ‘when teachers are given the opportunity and the resources, they welcome the challenges presented by GSD [gender and sexual diversity]' (Bryan, 2012, p. 133), this remains an area where schools continue to fall short based on Dean's experience.

Commendably, both policies offer a consideration of sex-segregated physical education (P.E.) classes and gender segregation in other classes where the policy insists that “students shall be permitted to participate in accordance with their gender identity.” However, such a policy that is *gender considerate* does not encourage schools and their educators to avoid gender segregation for the purpose of class activities and as an overall pedagogical strategy for addressing trans and non-binary inclusivity (Rands, 2009; Jackson, 2010; Ehrenhalt, 2016). Overall, there is clearly an emphasis on the logics of accommodation in both policies which appears to be motivated by and conceived in response to legislative requirements in the Ontario context with no significant allocation of resources and detailing of accountability measures for ensuring professional and curriculum development for teachers in schools.

## Interpretive Accounts of Trans-Inclusive Policies

In light of our focused analysis on the content and contextualization of these school board policies in Ontario, we draw on conversations from key informants to reflect upon and generate insights into trans-inclusive policy development and discursive limits and possibilities of policy frames that rely on a fundamental discourse of accommodation. We conceptualize the accounts that are derived from interviews with our participants as *snapshots* because they provide a window into the response to these policies by educators at a certain place and point in time. In this sense, they emphasize Ball's (1994) point about “policy as discourse” and as “set within a moving discursive frame which articulates and constrains the possibilities and probabilities of interpretation and enactment” (p. 2). It is in this sense that “the ‘effects' of policy cannot simply be read off from texts,” and that it is essentially how they are interpreted by actors in schools that is equally an important consideration in policy analysis (Ball, 1994, p. 21): “A policy is both contested and changing, always in a state of ‘becoming', of ‘was', and ‘not quite; ‘for any text a plurality of readers must necessarily produce a plurality of readings' (Codd, 1988, 239)” (Ball, 1994, p. 16).

### The Significance of Policy and Curriculum as Sites of Intervention

Each participant questioned whether policy was enough to foster more equitable conditions of access for transgender and gender diverse students in the public education system, echoing Spade's (2015) assertion that more is needed beyond the mere human rights legislative and policy frameworks. For example, participants discussed the idea about the potential of a trans-affirmative curriculum having a greater impact than the actual policy itself. Grace, a teacher of 5 years, was particularly optimistic about the current trans-affirmative policy, its current social relevance and the discussion surrounding it:

Well, we have to start from somewhere. Right? So right now, this is our starting point… It's current. People are talking about it …It's a good place to start talking …but it can't stay at that [trans-affirmative policy level] …It can't just remain a discussion of private enclosed places like the washroom …because it happens all the time that you get a topic that gets a lot of buzz and then *poof*, it's gone.

Grace affirmed that though the policy has surfaced during a “trans moment” (Nicolazzo, 2017b) with respect to transgender rights, it is crucial that the conversation regarding the importance of trans accommodation within schools is not seen as fulfilled simply because policy has created a space for discussing trans-affirmative engagement. As Kumashiro (2004) asserts:

… challenging oppression requires more than simply becoming aware of oppression, and this is because people are often invested in the status quo, as when people desire repeating what has become normalized in our lives. Change requires a willingness to step outside of this comfort zone (p. 46).

Therefore, policy itself is a necessary political intervention, but as Grace points out, it is rendered ineffective unless educators can address their own subconscious desires for learning and teaching within a gender binary and cisgenderist framework (Frohard-Dourlent, 2016; Smith and Payne, 2016; Morgan and Taylor, 2018). As Rands (2009) argues, a more gender-complex approach to education involves critically interrogating the *gender oppression matrix* as a basis for fostering professionally informed threshold knowledges about gender diversity. Thus, addressing gender democratization in the space of schools needs to be understood in terms of not just the official articulation of trans-affirmative policy discourse that relies on elaborating the specifics of accommodation, but of a concerted and long-term commitment on behalf of administrators and educators to interrogating institutionalized gender hierarchies and addressing cisgenderism (Connell, 2009; Nicolazzo, 2017c). By positioning accommodation requests as a resolution to trans marginalization in the education system, these policies tend to downplay the implications of requiring transgender students to surrender themselves to a process of investigation in order to receive permission to exist within a cisnormative system whilst refusing to restructure it. Moreover, it ignores Spade's (2015) cautioning to avoid such top-down approaches that do little to address more expansive equity issues.

Michael, for example, noted that Health and Physical Education is an important curriculum site where educators are required to officially address gender diversity at both the school board and Ministry of Education level (Ontario Ministry of Education, 2015):

… the Health and Phys-Ed curriculum that was just recently released […] is the only curricular expectations that speak specifically to gender identity and trans population. There are no other curricular expectations that lay that out specifically. So, how that looks in terms of how it's taken up in schools because it's still vague and wide enough to drive a truck through the way expectations are set up, again there's no P.D. that's been attached to it, no money that's been attached, so we'll see how that's embraced by Health and Phys-Ed teachers everywhere.

Addressing gender diversity with respect to physical education is important given the fact that this is an area where students are already interacting with themes of the body and imposed gender roles (Green, 2010). Michael's point, however, is that there are no allocated resources for professional development for teachers, and hence a lack of commitment on behalf of the school board despite its policy endorsement for supporting trans and gender diverse youth in schools. Moreover, he confirms that there are no specific curricular stipulations outside of the health and physical education curriculum, which is a fraught space, especially given the recent conservative government's regressive amendments to the 2015 version, which significantly delayed what grade educators are able to address gender and sexual diversity (Ferguson and Rushowy, 2019). Such contingencies further highlight the need for an officially sanctioned policy and curriculum framework as a support for teachers in schools with regards to addressing transgender and gender diversity on “both systematic and incidental levels” throughout the curriculum (Green, 2010, p. 6).

Michael, however, indicated that he has not witnessed any effort to employ a trans-inclusive curriculum by school administrators. Rather, he pointed out that the onus is on educators to create an accepting and safe learning environment that is encouraged in the trans-specific policy. In fact, Michael felt that steering a school to create an accepting learning environment with respect to embracing gender diverse expression in the classroom does not necessarily equate with developing a trans-informed curriculum:

I personally don't see that there's been any drive by the ministry to embed gender diversity education in the curriculum any more than it already is. There's kind of an emphasis in the Education Act that you're responsible for doing it, and it's something that's supposed to be done under the Accepting Schools Act that is sort of a daily… making sure that you're being inclusive, and respectful and all that sort of stuff. […] But I understand the nature of gender identity is not a learning outcome. [Laughs] In the curriculum, do I think that's going to happen anytime soon? I don't.

Despite the emphasis in the Education Act^3^ and the Accepting Schools Act^4^ 2012, evidence suggests that teachers are not effectively trained or provided with sustained professional development which explicitly addresses gender identity and gender complexity, and that this lack of training and the absence of trans-inclusive curriculum impact on enhancing understanding of trans inclusivity and livability in the school system (Luecke, 2011; Payne and Smith, 2014; Frohard-Dourlent, 2016; Smith and Payne, 2016; Goodrich and Barnard, 2018; Leonardi and Staley, 2018). Importantly, the presence of a trans-inclusive curriculum is significant given that in schools which had a curriculum that was LGBTQ-inclusive, students were less likely to hear negative remarks about transgender people (Kosciw et al., 2018, p. 70). Moreover, Michael exposes the limits of the policy in its failure to address resource allocation and accountability measures for supporting trans-affirmative curricular and professional development for principals and teachers in schools.

Grace also underscored this sentiment that teachers undoubtedly require further education: “Anybody who works in the school should have some sort of sensitivity training. We all do the workplace safety training.” When prompted about developing this understanding and education for teachers with respect to what form it would take, and who would run such a program, Grace answered simply: “P.D. Day [Professional Development Day]! We do everything online for WSIB [Workplace Safety and Insurance Board], stuff like that. I think it's possible to put together modules that you have to complete in order to stay employed. That's already been done. It's not a far stretch. Um. [Pause]. I mean, training will only do so much, but…” However, Green (2010) insists that these professional development days need to provide productive spaces for teachers to unpack understandings about gender diversity before they can adequately expect their students to do the same. Bryan (2012), for example, found that “teachers are quite blunt about the degree to which they already feel unsettled and unprepared when it comes to teaching about gender and sexual diversity” (p. 133) (Payne and Smith, 2014; Ullman, 2015; see also Leonardi and Staley, 2018). Therefore, it is clear that some teachers are willing to combat their feelings of unpreparedness by educating themselves on gender diversity in order to create an inclusive classroom environment for their students. However, Smith and Payne (2016) found that after attending trans-informed professional development, many teachers “resisted gender-affirming pedagogy and fixated on the logistics of accommodating transgender students and keeping them safe” (p. 34), which speaks to the logics at the heart of the school board policies that are the subject of this article.

Nevertheless, Michael stressed that the responsibility for ensuring respect for diversity rests with the school's code of conduct. He added that while the latter is important, without proper education for teachers regarding issues of *gender sensitivity* and the need for a pedagogical commitment to addressing gender diversity and trans inclusivity more broadly, students have a difficult time understanding the extent to which compulsory heterogenderism^5^ and cisgender systems actually operate to deny trans recognizability and livability in the school system (Wyss, 2004; Taylor et al., 2011):

[The code of conduct] is supposed to inform students about how they should be behaving, and when they don't behave that way, they get punished. So, we've really set up the system terribly in the sense that staff who are expected to give the message haven't been properly trained. There's no focus on what that training should look like for staff in a regular curriculum day. There's no emphasis of the priority for that within the curriculum itself. And students who need the information to be able to understand how to create a respectful environment don't necessarily get it from the staff—because they haven't received the training—get punished when they don't behave that way.

Michael suggests that simply writing the expectation of respect into the code of conduct is insufficient, and he points to the limits of a liberal focus on diversity as a basis for educating about trans inclusivity and addressing the specific needs of gender minority students (Gressgård, 2010; Martino and Cumming-Potvin, 2015). Rather, what is required, he argues, is a focus on *why* this respect is essential in terms of ensuring gender democratization and gender expansive understandings in the education system (Pyne, 2014; Martino and Cumming-Potvin, 2018). Moreover, such an assertion highlights the significance of embracing a model of *trickle up* social justice that shifts the focus away from merely accommodating individual students to one that embraces a systemic consideration of trans marginalization in the education system with its attention to creating self-determining spaces for trans and non-binary students to be recognized and affirmed (Spade, 2015; Frohard-Dourlent, 2018). Specifically, addressing the concerns of one student at a time does little to restructure a problematic cisgenderist system. Our policy critique in fact highlights that there needs to be a move beyond such reactive approaches to the presence of trans students in schools to encourage a broader focus on gender diversity as a necessary basis for addressing trans affirmative education (see also Frohard-Dourlent, 2018). This critical insight has vital implications for policy formulation and frames with respect to moving beyond an individualistic focus on accommodating the tans student. For example, research by Taylor et al. (2011) in Canada and Kosciw et al. (2018) in the United States found that trans students felt more comfortable and a greater sense of belonging in schools with specific LGBTQ inclusive policies and curriculum (p. 79). Our policy analysis is informed by such empirical insights and, hence, speaks to our critique of policy frames that eschew a much-needed focus on trans informed curriculum development and pedagogical intervention. Such a redirected focus highlights the need for more systemic education about gender diversity as opposed to more a reactive approach to relying merely on the presence of a trans student as basis for instigating gender diversity education. In fact, while Meyer et al.'s (2016) research with teachers revealed that the presence of a trans or gender-creative student was instrumental in initiating intervention and support measures with respect to addressing trans inclusion, they are critical of what they refer to as “a pedagogy of exposure” where the individual trans student risks becoming the “sacrificial lamb” for instigating more systemic policy enactment and curricular intervention designed to address and educate about gender diversity (p. 17). This does not mean that trans and non-binary students should not be at the center of trans affirmative policy articulation and enactment in schools. In fact, it is vital that trans-informed policies prioritize “building leadership and membership on a “most vulnerable first” basis, centering the belief that social justice trickles up, not down and that meaningful change comes from below” (Spade, 2015, p. 137). However, intervention and gender justice education which is taken solely in response to the presence of the trans student or by trans students themselves advocating for themselves and for such education constitutes a fundamental abnegation of responsibility on behalf of the education system to ensure the safety, privacy and well-being of gender diverse youth. Through an approach which entails school boards and schools actively supporting and taking responsibility for gender complex education (Rands, 2009), constructive steps toward gender democratization which entails a “shift [in] focus from the individual rights framing of discrimination …. [to] think[ing] more broadly about how gender categories are enforced on all people in ways that have particularly dangerous outcomes for trans people” can be actualized (Spade, 2015, p. 9).

In order to move toward gender democratization, participants appeared to advocate for what Rands (2009) refers to as a gender-complex approach to education which entailed raising awareness about regimes of *compulsory heterogendersim* and cisnormativity with their occlusion of trans and othering of non-binary subjectivities (Ehrenhalt, 2016; Worthen, 2016; Nicolazzo, 2017c) All three participants underscored the importance of developing a trans-affirmative curriculum which moved beyond merely accommodating trans students. However, they indicated that they were not aware of systematic professional development devoted to addressing gender identity and trans-inclusive education despite the School Board A's policy support for such initiatives, thereby foregrounding the lack of any real commitment to resource allocation to achieve these ends.

### The Limits of Accommodation

The participants agreed that despite each school board policy's emphasis on accommodation, they did not appear to translate into fostering a safe space for trans students. Grace, for example, insisted “that the real weakness is the accommodation based on request” aspect of their school board policy. When asked to elaborate on why they perceived this to be the case, their answer echoed those of the other participants:

I think that it creates a bit of a problem in that a student—anybody—might know that you don't fit female, but they don't really know if they want to fit into male. So, having that binary there established and saying, “Well, you have to fit into one of these and if you don't, you have to out yourself” when you might not even know what that means yet. Right? So, knowing that you're not the same as a binary isn't the same as knowing definitively, “I identify as trans.” “I identify as queer.”

Having students feel that they must *out* themselves in order to be accommodated has the potential to increase surveillance of trans and non-binary bodies and, hence, enhances the very risk of being victimized (Ingrey, 2018). Moreover, by placing the onus on students to request their required accommodation, presumes that students who are, as Grace suggests, non-binary, understand what kind of accommodation(s) they require. In this respect, policies and schools need to envision a “transgender imaginary” which “encapsulates more dynamic possibilities in the realization of gendered personhood” (Martino, 2016, p. 383). This is a significant approach that is “grounded necessarily in the voices and embodied experiences of trans subjects themselves,” and must be understood in response to what Namaste (2000) documents as “the epistemic violence that has contributed to the institutional and cultural erasure of the lived and bodily ontological existence of transgender people in the everyday world” (p. 382). Policies must offer a more nuanced consideration of the spectrum of embodiment and how reactionary accommodation based upon request is not as straightforward for all transgender and gender diverse individuals but also need to commit to resource allocation to foster more gender expansive education in schools.

This notion of requiring a student to *out* themselves based upon their gender identity and their need for accommodation is paradoxical to the very creation of the policies themselves, as both policies cite the Taylor et al. (2011) report that documented the alarming statistics of trans student victimization occurring within schools. Grace, for instance, linked the limits of accommodation to this potential for increased victimization and marginalization:

It [the policy] asks people to *out* themselves and mark themselves as different, which then puts them at a higher risk of being victimized. […] I think that different people might find different solutions. … I would hope that there's somebody they can talk to and… “Based on request”—I don't know if it says it in here if it has to be the actual student who makes the request. Because having a friend ask would be a solution as well. I don't know if it would be possible to anonymously ask or make a request. But it is a barrier in, you know, receiving the accommodations that are promised in this.

By placing the onus on the students to not only out themselves but also claim their own transgender identity and the subsequent required accommodations, these policies continue to enforce cisgender privilege in schools. While still maintaining the dominant gender binary, the policies create an “other” gender category, in which a student must situate themselves if they are not cisgender. The creation of an “other” gender category, as Namaste (2000) further explains, “allows for a transgender identification but also denies a simultaneous identification with the gender of ‘man' or ‘woman', while collapsing the different ways of identifying as transgendered and living one's life” (p. 44).

This system of having trans or gender diverse students declare their embodied differences reinforces the gender oppression matrix of which Rands (2009) speaks, which fails to “take into consideration those who do not identity within the binary gender categorization of men/boys and women/girls” (p. 423). As a result, while transgender students are not absolutely stripped of the right to use a bathroom that corresponds with their gender identity, they are denied the right of entering whichever bathroom they feel comfortable by having to *request* to be accommodated.

An emphasis on accommodation based upon individual request invokes no substantive change to the cisnormative system. As Dean explains: “I was away for 2 years, right? And so, you'd think if things had shifted [due to the policy], I would have noticed a difference. And I don't notice much of a difference.” The polemic of relying on singular accommodations as opposed to invoked more sweeping proactive systemic interventions does little to interrogate or dismantle the cisnormative system, but instead requires individual students to submit to interrogation. In this respect, schools must heed Greytak et al.'s (2009) and Taylor et al.'s (2011) invitation to become proactive in addressing systemic issues by actively promoting and suffusing trans-affirmative resources, curriculum, and pedagogy within schools which ensure “the climate is significantly more positive for sexual and gender minority students” (Taylor et al., 2011, p. 28).

One of the unintended effects of the “accommodation based upon request” invoked by these policies is that proactive interventions may not be undertaken until they are sought out by trans students themselves. Mathers (2017), for example, highlights the problem of the cisnormative dynamics at play in how binary understandings of gender are mobilized to “make sense of transgender experience while placing an unequal emotional burden on transgender and gender non-conforming people to mend the interactional disruption of the gender panic” (p. 295). This polemic of relying on a policy discourse of accommodation at the expense of an emphasis on the necessity of creating and resourcing the pedagogical conditions for educating about gender diversity highlights the ethical, epistemic and political considerations at play in addressing the articulation of trans-affirmative policies in the education system (Journell, 2017; Mangin, 2018).

## Conclusion and Implications

In this article our focus has been on generating critical insights into the development of trans-affirmative policies and practices in specific school boards/school contexts, given the dearth of research that exists on this topic (Jones et al., 2016; Bartholomaeus and Riggs, 2017; Neary, 2018; Martino and Cumming-Potvin, 2019). Our case study enabled us to provide some particularity and context specificity about the formulation of trans inclusive policies in the Ontario context, with the objective of generating knowledge about the limitations of relying on a discourse of accommodation as a basis for supporting transgender and gender diverse students in the education system. Indeed, our research has highlighted that there is a continued need for educators and administrators to pay close attention to ethical and political questions of trans livability in education and school systems as part of an overall project of fostering gender justice and gender democratization for all students (Rands, 2009; Pyne, 2014). It has also foregrounded the necessity of trans-affirmative policy that is committed to addressing trans marginalization with respect to the provision of in-service and pre-service education to ensure that requisite teacher threshold knowledges about gender diversity and cisgenderism can be enacted beyond merely appealing to a fundament imperative of accommodation (Rands, 2009; Luecke, 2018). As Michael—equity officer with one of the school boards where we conducted our research—pointed out, resources are needed to support schools and teachers in this endeavor and political project of enacting gender democratization. This particular school board has a team of educators and social workers who are equipped with knowledge and expertise and who are sought out by schools to support administrators, educators and students in terms of enacting the policy with respect to its stipulations for accommodating trans students and addressing complex education. In this respect, there is some provision of trans-informed professional development and support for schools. However, there is a necessity for policymakers to explicitly address resource allocation and accountability more systematically. Moreover, such support and education must extend beyond merely fulfilling the accommodation terms of the policy, which requires and holds the board legally responsible for a failure to “respond to a transgender student's concerns or request” (Ludeke, 2009, p. 16).

Finally, in addition to staff and administrators requiring a deeper understanding of trans marginalization, this study has actively troubled the requirement bestowed upon trans and gender diverse students to request accommodation. It is important that schools deeply consider the necessity of students safely accessing these gender-segregated areas without having to request to do so. Putting the onus on the individual trans student in this respect translates into a fundamental abnegation of the education system's responsibility to actively address the broader cisgenderist forces at play and their institutionalization in schools which make it difficult not only for trans youth to navigate the system on daily basis, but to feel comfortable about being out and visible in the first place, a phenomenon which (Carlile and Paechter, 2018) refer to as “precarious invisibility” (p. 86) (see also Bartholomaeus and Riggs, 2017; Ferfolja and Ullman, 2020). In this sense, trans-affirmative policy ultimately needs to engage in a sustained way with a critical trans politics that is committed to both “conceptualiz[ing] the conditions trans people face and more directly strategiz[ing] change that impacts the well-being of trans people” (Spade, 2015, p. 16).

## Data Availability Statement

The datasets generated for this study are available on request to the corresponding author.

## Ethics Statement

The studies involving human participants were reviewed and approved by The Office of Human Research Ethics (OHRE), on behalf of Western's Research Ethics Boards (REB) at the University of Western Ontario. The patients/participants provided their written informed consent to participate in this study.

## Biographical Notes

KO is a Ph.D. student in the Faculty of Education at the University of Western Ontario in London, Ontario, Canada. His research interests center upon examining the structural and political obstacles surrounding the consideration of transgender and gender diverse perspectives in the education system. WM is a professor of Equity and Social Justice Education in the Faculty of Education and an affiliate member of the Department of Women's Studies and Feminist Research at the University of Western Ontario, Canada. His research interests include addressing queer and transgender informed perspectives on gender justice and democratization in the education system.

## Author Contributions

KO was responsible for the design of the study and conducting the interviews with the key informants for the research study and conducted the thematic analysis and coded the data accordingly. KO and WM collaboratively conducted the proper ethics protocols and engaged in the writing process of the article together. WM draws significantly on the application of trans epistemological insights and approaches to critical policy analysis that are derived from his SSHRC funded research into supporting trans youth in schools.

## Conflict of Interest

The authors declare that the research was conducted in the absence of any commercial or financial relationships that could be construed as a potential conflict of interest.

## Footnotes

^1^All school boards in Ontario are required to ensure that they meet the Ontario Ministry of Education's equity stipulations in accordance with the Ontario Human Rights Code. While provincially developed policies are to be enforced by all school boards in the province, the policies developed by the school board are only implemented by schools that fall within that board's jurisdiction.

^2^There is no Federal Department of Education in Canada. Educational policy in Canada is developed provincially and is specific to each provincial jurisdiction. Ontario is comprised of three branches of government: legislative, executive and judicial. The executive branch is comprised of elected Members of Provincial Parliament (MPPs) who introduce policy for consideration in the House of Commons. The decisions made in Cabinet provide direction for policy development and implementation in the Ontario Public Service (OPS). The OPS is comprised of non-partisan staff who develop and implement policy. The development of such policies can be instigated through the proposal of a bill by an MPP that may be encouraged by public opinion.

^3^The Education Act was amended in 2012 by Bill 13 to formally mandate the promotion of awareness, safety, and inclusion of transgender students in schools in order to prevent transphobia.

^4^The *Accepting Schools Act* requires schools to prevent and address inappropriate and disrespectful behaviour among students in schools. It requires schools to have policies in place that address bullying and ensure inclusive education. The passage of the act controversially allowed for the creation of Gay Straight Alliances without being vetoed or disallowed in public schools in Ontario.

^5^Nicolazzo (2017c) defines *compulsory heterogenderism* as “a cultural condition by which diverse gender identities are positioned as abject or culturally unintelligible” which contributes to their erasure and “makes one's gender identity incomprehensible, unknowable, and invalid” (p. 247).

## References

[B1] Accepting Schools Act (2012). SO 2012. c. 5. Available online at: https://www.ontario.ca/laws/statute/S12005

[B2] Ashley F. (2018). Don't be so hateful: the insufficiency of anti-discriminatory hate-crimes laws in improving trans wellbeing. Univ. Toronto Law J. 68, 1–36. 10.3138/utlj.2017-0057

[B3] Bacchi C. L. (2000). Policy as discourse: what does it mean? Where does it get us? Discourse 21, 45–57. 10.1080/01596300050005493

[B4] Bacchi C. L. (2009). Analysing Policy: *What's the Problem Represented to Be?* Frenchs Forest, NSW: Pearson.

[B5] Bacchi C. L. Eveline J. (2010). Mainstreaming Politics: Gendering Practices and Feminist Theory. University of Adelaide Press.

[B6] Ball S. J. (1993). What is policy? Texts, trajectories, and toolboxes. Discourse 13, 10–17. 10.1080/0159630930130203

[B7] Ball S. J. (1994). Education Reform. Buckingham: Open University Press.

[B8] Bartholomaeus C. Riggs D. W. (2017). Transgender People and Education. New York, NY: Palgrave Macmillan. 10.1057/978-1-349-95309-7

[B9] Bill C-16 (2016). An Act to Amend the Canadian Human Rights Act and the Criminal Code, 1st sess. 42nd Parliament.

[B10] Braun A. Maguire M. Ball S. J. (2010). Policy enactments in the UK secondary school: examining policy, practice and school positioning. J. Educ. Policy 25, 547–560. 10.1080/02680931003698544

[B11] Bryan J. (2012). From the Dress-Up Corner to the Senior Prom: Navigating Gender and Sexuality Diversity in PreK-12 Schools. Lanham, Md: Rowman & Littlefield Education.

[B12] Carlile A. (2019). Teacher Experiences of LGBTQ- Inclusive Education in Primary Schools Serving Faith Communities in England, UK. Pedagogy, Culture and Society, 1–20. 10.1080/14681366.2019.1681496

[B13] Carlile A. Paechter C. (2018). LGBTQI Parented Families and Schools: Visibility, Representation and Pride. New York, NY; Abingdon, Oxon: Routledge. 10.4324/9781315674148

[B14] Clarke V. Braun V. (2017). Thematic analysis. J. Posit. Psychol 12, 297–298. 10.1080/17439760.2016.1262613

[B15] Codd J. (1988). The construction and deconstruction of educational policy documents. J. Educ. Policy 3, 235–248. 10.1080/0268093880030303

[B16] Connell R. (2009). Short Introductions: Gender. Cambridge: Polity Press.

[B17] Courvant D. (2011). Strip! The Radical Teacher. New York, NY: Center for Critical Education of New York, 26–34. 10.5406/radicalteacher.92.0026

[B18] Cumming-Potvin W. Martino W. (2018). The policyscape of transgender equality and gender diversity in the western australian education system: a case study. Gend. Educ 30, 715–735. 10.1080/09540253.2018.1483491

[B19] Egale (2012). Report on Outcomes and Recommendations: LGBTQ Youth Suicide Prevention Summit. Report. Availabe online at: https://egale.ca/wp-content/uploads/2013/02/YSPS-Report-online.pdf

[B20] Ehrenhalt J. (2016). Being There for Nonbinary Youth. Teaching Tolerance. Availble online at: https://www.tolerance.org/magazine/summer-2016/being-there-for-nonbinary-youth

[B21] Ferfolja T. Ullman J. (2020). Gender and Sexuality Diversity and a Culture of Limitation: Student and Teacher Experiences in Schools. New York and Abingdon, Oxon: Routledge.

[B22] Ferguson R. Rushowy K. (2019). Ford Government's Sex-Ed Changes Blasted From All Sides. Toronto Star. Retrieved from: https://www.thestar.com/politics/provincial/2019/08/21/premier-doug-ford-governments-sex-ed-changes-blasted-by-former-pc-leadership-candidate.html

[B23] Frohard-Dourlent H. (2016). ‘I don't care what's under your clothes': the discursive positioning of educators working with trans and gender-nonconforming students. Sex Educ. 16:63. 10.1080/14681811.2015.1022819

[B24] Frohard-Dourlent H. (2018). ‘The student drives the car, right?': Trans students and narratives of decision-making in schools. Sex Educ. 18, 328–344. 10.1080/14681811.2017.1393745

[B25] Goodrich K. Barnard J. (2018). Transgender and Gender Non-Conforming Students in Schools: One School District's Approach for Creating Safety and Respect. Sex Education, 1–14. 10.1080/14681811.2018.1490258

[B26] Green E. R. (2010). Shifting paradigms: moving beyond trans 101 in sexuality education. Am. J. Sex. Educ 5, 1–16. 10.1080/15546121003748798

[B27] Gressgård R. (2010). When trans translates into tolerance - or was it monstrous? Transsexual and transgender identity in liberal humanist discourse. Sexualities 13, 539–536. 10.1177/1363460710375569

[B28] Greytak E.A. Kosciw J.G. Diaz E. M. (2009). Harsh Realities: The Experiences of Transgender Youth in Our Nation's Schools. New York, NY: GLSEN.

[B29] Human Rights Campaign Gender Spectrum (2018). Children's Books on Gender and Transgender & Non-Binary Students | Welcoming Schools. Available online at: http://www.welcomingschools.org/resources/books/transgender-youth/

[B30] Ingrey J. (2018). Problematizing the cisgendering of school washroom space: interrogating the politics of recognition of transgender and gender non-conforming youth. Gend. Educ. 30, 774–789. 10.1080/09540253.2018.1483492

[B31] Jackson J. (2010). ‘Dangerous presumptions': how single-sex schooling reifies false notions of sex, gender, and sexuality. Gend. Educ. 22, 227–238. 10.1080/09540250903359452

[B32] Jones T, Smith, E. Ward R. Dixon J. Hillier L. Mitchell A. (2016). School experiences of transgender and gender diverse students in Australia. Sex Educ. 16, 156–171. 10.1080/14681811.2015.1080678

[B33] Journell W. (2017). Framing controversial identity issues in schools: the case of HB2, bathroom equity, and transgender students. Equity Excell. Educ. 50, 339–354. 10.1080/10665684.2017.1393640

[B34] Keenan H. B. (2017). Unscripting curriculum: toward a critical trans pedagogy. Harv. Educ. Rev. 87, 538–556. 10.17763/1943-5045-87.4.538

[B35] Kirkup K. (2018). The origins of gender identity and gender expression in anglo-american legal discourse. Univer. Toronto Law J. 68, 80–117. 10.3138/utlj.2017-0080

[B36] Kjaran J. I. (2019). Gender-segregated spaces in icelandic high schools: resistance, power and subjectification. Gend. Educ. 31, 1020–1039. 10.1080/09540253.2017.1401046

[B37] Kosciw J, Greytak, E. Zongrone A. Clark C. Truong N. (2018). The 2017 National School Climate Survey The 2017 National School Climate Survey The Experiences of Lesbian, Gay, Bisexual, Transgender, and Queer Youth in Our Nation's Schools. New York, NY: GLSEN.

[B38] Kumashiro K. K. (2004). Against Common Sense: Teaching and Learning Toward Social Justice. New York, NY: Routledge Falmer.

[B39] Lennon E. Mistler B. J. (2014). Cisgenderism. Transg. Studies Quart. 1, 63–64. 10.1215/23289252-2399623

[B40] Leonardi B. Staley S. (2018). What's involved in 'the work'? Understanding administrators' roles in bringing trans-affirming policies into practice. Gend. Educ. 30:754. 10.1080/09540253.2018.1455967

[B41] Ludeke M. (2009). Transgender Youth. Vol. 10. Reston: National Association of Secondary School Principals.

[B42] Luecke J. C. (2011). Working with transgender children and their classmates in pre-adolescence: just be supportive. J. LGBT Youth 8, 116–156. 10.1080/19361653.2011.544941

[B43] Luecke J. C. (2018). The gender facilitative school: advocating authenticity for gender expansive childre in pre-adolescence. Improv. Schools 2, 269–264. 10.1177/1365480218791881

[B44] Malatino H. (2015). Pedagogies of becoming: trans inclusivity and the crafting of being. TSQ: Transgender Studies Quarterly 2, 395–410. 10.1215/23289252-2926387

[B45] Mangin M. (2018). Supporting transgender and gender-expansive children in school. Phi Delta Kappan 100:16. 10.1177/0031721718803564

[B46] Martino W. Airton L. Kuhl D. Cumming-Potvin W. (2019). Mapping transgender policyscapes: a policy analysis of transgender inclusivity in the education system in Ontario. J. Educ. Policy 1–29. 10.1080/02680939.2018.1478992

[B47] Martino W. Cumming-Potvin W. (2018). Transgender and gender expansive education research, policy and practice: reflecting on epistemological and ontological possibilities of bodily becoming. Gend. Educ. 30, 687–694. 10.1080/09540253.2018.1487518

[B48] Martino W. Cumming-Potvin W. (2019). ‘Effeminate arty boys and butch soccer girls': investigating queer and trans-affirmative pedagogies under conditions of neoliberal governance. Res. Papers Educ. 34, 131–152. 10.1080/02671522.2017.1402082

[B49] Martino W. J. (2016). The transgender imaginary, in Critical Concepts in Queer Studies and Education, eds Rodriguez N. M. Martino W. J. Ingrey J. C. Brockenbrough E., (New York, NY: Palgrave Macmillan), 381–394. 10.1057/978-1-137-55425-3_37

[B50] Martino W. J. Cumming-Potvin W. (2015). Teaching about ‘princess boys' or not: the case of one male elementary school teacher and the polemics of gender expression and embodiment. Men Masc. 18, 79–99. 10.1177/1097184X14551278

[B51] Martino W. J. Cumming-Potvin W. (2016). Teaching about sexual minorities and princess boys: a queer and trans-infused approach to investigating LGBTQ-themed texts in the elementary school classroom. Discourse 37, 807–827. 10.1080/01596306.2014.940239

[B52] Mathers L. A. B. (2017). Bathrooms, boundaries, and emotional burdens: cisgendering interactions through the interpretation of transgender experience. Symb. Inter. 40, 295–316. 10.1002/symb.295

[B53] Meyer E. J. Tilland-Stafford A. Airton L. (2016). Transgender and gender-creative students in PK-12 schools: what we can learn from their teachers. Teach. Coll. Rec 118, 1–50.

[B54] Millei Z. Cliff K. (2014). The preschool bathroom: making ‘problem bodies' and the limit of the disciplinary regime over children. Br. J. Sociol. Educ 35, 244–262. 10.1080/01425692.2012.761394

[B55] Morgan E. Taylor Y. (2018). Dangerous education: the occupational hazards of teaching transgender. Sociology 53, 19–35. 10.1177/0038038517746052

[B56] Namaste V. (2000). Invisible Lives: The Erasure of Transsexual and Transgendered People. Chicago, IL: University of Chicago Press.

[B57] Neary A. (2018). New trans visibilities: working the limits and possibilities of gender at school. Sex Educ. 18, 435–448. 10.1080/14681811.2017.1419950

[B58] Nicolazzo Z. (2017a). Imagining a Trans Epistemology: What Liberation Thinks Like in Postsecondary Education. Urban Education, 1–26. 10.1177/0042085917697203

[B59] Nicolazzo Z. (2017b). Introduction: what's transgressive about trans studies in education now? Int. J. Qual. Studies Educ. 30:211. 10.1080/09518398.2016.1274063

[B60] Nicolazzo Z. (2017c). Compulsory heterogenderism: a collective case study. NASPA J. Women Higher Educ. 10, 245–261. 10.1080/19407882.2017.1351376

[B61] Ontario Human Rights Commission (2012a). The Ontario Human Rights Code. Ontario Human Rights Commission. Available online at: www.ohrc.on.ca/en/ontario-human-rights-code (accessed August 31, 2018).

[B62] Ontario Human Rights Commission (2012b). Gender Identity and Gender Expression. Availble online at: http://www.ohrc.on.ca/en/gender-identity-and-gender-expression-brochure (accessed November 28, 2019).

[B63] Ontario Ministry of Education (2015). Sex Education in Ontario. Government of Ontario. Available online at: https://www.ontario.ca/page/sex-education-ontario (accessed August 31, 2018).

[B64] Patton M. Q. (2015). Qualitative Research & Evaluation Methods: Integrating Theory and Practice. Fourth ed. Thousand Oaks, CA: SAGE.

[B65] Payne E. Smith M. (2014). The big freak out: educator fear in response to the presence of transgender elementary school students. J. Homosex 61, 399–418. 10.1080/00918369.2013.842430 24397377

[B66] Phillips D. Ochs K. (2004). Researching policy borrowing: some methodological challenges in comparative education. Br. Educ. Res. J. 30, 773–784. 10.1080/0141192042000279495

[B67] Prosser J. (1998). Second Skins: The Body Narratives of Transsexuality. New York, NY: Columbia University Press.

[B68] Pyne J. (2014). Gender independent kids: a paradigm shift in approaches to gender non-conforming children. Can. J. Hum. Sex 23, 1–8. 10.3138/cjhs.23.1.CO1

[B69] Rands K. E. (2009). Considering transgender people in education: a gender-complex approach. J. Teach. Educ. 60, 419–431. 10.1177/0022487109341475

[B70] Ryan C. L. Patraw J. M. Bednar M. (2013). Discussing princess boys and pregnant men: teaching about gender diversity and transgender experiences within an elementary school curriculum. J. LGBT Youth 10, 83–105. 10.1080/19361653.2012.718540

[B71] Shanks D. Lester J. (2019). How to Accommodate Transgendered or Gender-Nonconforming Students in Schools. Available online at: https://www.cheadles.com/human-rights-law/accommodate-transgendered-gender-nonconforming-students-schools/ (accessed December 31, 2019).

[B72] Sinclair J. Gilbert J. (2018). Naming new realities: supporting trans youth in education. Sex Educ. 18, 321–327. 10.1080/14681811.2018.1452347

[B73] Slater J. Jones C. Procter L. (2018). School toilets: queer, disabled bodies and gendered lessons of embodiment. Gend. Educ. 30, 951–965. 10.1080/09540253.2016.1270421

[B74] Smith M. J. Payne E. (2016). Binaries and biology: conversations with elementary education professionals after professional development on supporting transgender students. Educ. Forum 80, 34–47. 10.1080/00131725.2015.1102367

[B75] Spade D. (2015). Normal life: Administrative Violence, Critical Trans Politics, and The Limits of Law. Brooklyn, NY: South End Press. 10.1215/9780822374794

[B76] Stryker S. (2006). (de)subjugated knowledges: an introduction to transgender studies, in The Transgender Studies Reader, eds Stryker S. Whittle S. (New York, NY: Routledge), 1–18.

[B77] Stryker S. Aren A. Z. (2013). Introduction: transgender studies 2.0, in The Transgender Studies Reader 2, eds Stryker S. Aizura A. Z. (New York, NY: Routledge), 1–12. 10.4324/9780203955055

[B78] Taylor C. G. Peter T. EGALE Canada Human Rights Trust. (2011). Every Class in Every School: Final Report on The First National Climate Survey on Homophobia, Biphobia, and Transphobia in Canadian Schools. Toronto: Egale Canada Human Rights Trust.

[B79] Ullman J. (2015). Ladylike/butch, sporty/dapper: exploring ‘gender climate' with Australian LGBTQ students using stage–environment fit theory. Sex Educ. 14, 430–443. 10.1080/14681811.2014.919912

[B80] Ullman J. (2017). Teacher positivity towards gender diversity: exploring relationships and school outcomes for transgender and gender-diverse students. Sex Educ. 17, 276–289. 10.1080/14681811.2016.1273104

[B81] Worthen M. G. F. (2016). Hetero-Cis-normativity and the gendering of transphobia. Int. J. Transgender. 17, 31–57. 10.1080/15532739.2016.1149538

[B82] Wyss S. E. (2004). ‘This was my hell': the violence experienced by gender non-conforming youth in US high schools. Int. J. Q. Studies Educ. 17, 709–730. 10.1080/0951839042000253676

[B83] Youth Gender Action Project (2009). Trans Youth at School: Y-Gap Community Bulletin. Available online at: http://www.ctys.org/wp-content/uploads/2013/06/YGAP_School.pdf

