# Erratum: Supporting Transgender Inclusion and Gender Diversity in Schools: A Critical Policy Analysis

**DOI:** 10.3389/fsoc.2020.00056

**Published:** 2020-07-28

**Authors:** Frontiers Production Office

**Affiliations:** Frontiers Media SA, Lausanne, Switzerland

**Keywords:** gender justice, gender democratization, trans-affirmative policy, transgender, trans inclusion, gender diversity, transgender students, non-binary

Due to a production error, “(Shanks and Lester, 2019)” was incorrectly written as “(Shanks and Lester, 1994).” The citation has been updated throughout the article and in the reference list.

The publisher apologizes for this mistake. The original article has been updated.
